# Registration of ovarian cancer in England and Wales

**DOI:** 10.1054/bjoc.2000.1255

**Published:** 2000-06-15

**Authors:** M J Quinn, B Botting, D Foote, A Read

**Affiliations:** National Cancer Intelligence Centre; Medical Research Services; NHSCR Office for National Statistics


British Journal of Cancer (2000) 83(2), 278–279
© 2000 Cancer Research Campaign
doi: 10.1054/ bjoc.2000.1255, available online at http://www.idealibrary.com on IDEAL®

Letter to the Editor

### Registration of ovarian cancer in England and Wales

**Sir**,
It is not true that ovarian cancer is more than 20% under-registered in England and Wales as alleged by Macdonald et al (1999).

For the vast majority of cohort studies which use the National Health Service Central Register (NHSCR) in Southport (part of the Office for National Statistics (ONS)) to obtain information on cancer or death, the individuals are 'flagged' (i.e. permanently marked on the register) so that any existing and all future data will be notified to the researchers. Another process called 'follow up' is also possible when subjects are checked against the register on a single occasion. This of course, is considerably cheaper than flagging, but has the disadvantage that at any one point in time the cancer information may be incomplete owing to the inevitable time lags and/or backlogs in the system of cancer registration.

There are three basic reasons why cases of cancer may not have been notified to researchers at a particular point in time:

1 *The case is not (or not yet) registered* The case may have been missed by the relevant regional cancer registry – either when the person was alive, or if the person died without cancer being mentioned on the death certificate (in which case a notification of the death would not have been sent from ONS to the registry (ONS 1999)). Alternatively, the registry could still be trying to find out when the cancer was first diagnosed.
2 *The case is registered but not yet flagged at NHSCR* The case may have been recorded by the regional cancer registry, but not forwarded to the National Cancer Intelligence Centre (NCIC) at ONS Titchfield (perhaps because not all the required data items were present). Or though sent to the NCIC, validation checks revealed errors which required returning to the registry for correction. Other delays can occur when records do not match automatically on the central register at Southport and require operator attention.
3 *The case is untraced or mistraced at NHSCR* In the past, about 3% of records could not be traced, but with recent modifications both at NHSCR and regional cancer registries, the rate has now fallen to below 1%. The number of mis-matches is extremely small, and usually only occurs when the name is a very common one.

Despite several attempts by ONS staff to explain these problems and to persuade MacDonald et al to have their cohort flagged so that they would, eventually, get all the available information, they insisted on follow up only. Some of the above factors particularly affected the ovarian screening study.

First, a large proportion of the women in the study lived in the south east of England. Until quite recently, the methods of registration used by the Thames Cancer Registry which covers this area (about a quarter of the population of England) resulted in about 20% of registrations overall being made solely from the information on a death certificate (DCO). In 1996, for example, the DCO rate was 24% overall; for ovarian cancer it was 27% (Thames Cancer Registry, 1997). As 5 year survival for ovarian cancer is around 30%, it is likely that there was under-registration of around 10% (Parkin et al, 1994). The proportion of ovarian cancer records for the rest of England and Wales with zero survival (a mixture of DCO registrations and true zero survival as a result of findings at post mortem) is under 5% so under-registration outside the Thames regions must be very small.

Second, in 1997 when the researchers first notified us of a shortfall in the information sent to them, not all of the registrations for 1992 and 1993 had been received at ONS. Indeed, the data for 1993 were judged to be complete only in July of last year (1999).

Third, in 1997, there were backlogs in the flagging of cancers at the NHSCR owing to the high priority work for the Department of Health of introducing the new NHS numbers. It was only in April 1998 that flagging was complete for cases received up to 1991 incidence year. But by September 1999 NHSCR had caught up and had flagged all the available cases for 1996 (then about half the expected eventual total).

In addition, staff at the NHSCR were unable to flag many members of the cohort because the information supplied by the researchers was inadequate – for example, lack of full, and accurate, date of birth.

For the reasons given above, MacDonald et al's analysis should have been restricted to cases diagnosed before 1991. Of seven such alleged cases not previously notified to the researchers which were investigated by ONS in early 1998, two were traced with a cancer registration (despite both dates of birth supplied by the researchers being incorrect), and one (with a Scottish address) was traced by the Scottish NHSCR, leaving only four cases – all of which, on further checking, were not on the relevant regional cancer register (three were resident in the Thames regions). At that time, MacDonald et al said that they had been notified by NHSCR of four cases (two post-1990) not previously known to them (rather than the figure of two given in their paper).

It is disappointing that following initial collaboration with ONS, and strenuous efforts made by our staff to find cases in advance of the scheduled processing, MacDonald et al chose to publish their paper without even letting us know that they were doing so, let alone checking their data and conclusions with us. We stress that lack of notification of cases to researchers does not necessarily mean under-registration.

Justified criticisms based on adequate data and taking account of the relevant circumstances – particularly if accompanied by constructive suggestions for improvements to our services – are always welcome. The publication of exaggerated conclusions based on small numbers of cases without consideration of all the relevant factors serves only to damage the system of cancer registration in England and Wales and unfairly bring into question the quality of the work of the cancer registries and ONS.

*Dr M J Quinn, Director, National Cancer Intelligence Centre*
*Mrs B Botting, Co-ordinator, Medical Research Services*
*Mr D Foote, Manager, National Cancer Intelligence Centre*
*Mr A Read, Manager of NHSCR*
*Office for National Statistics*

**REFERENCES**

Macdonald N, Sibley K, Rosenthal A, Menon U, Jeyarajah A, Oram D and Jacobs I (1999) A comparison of national cancer registry and direct follow-up in the ascertainment of ovarian cancer. *Br J Cancer* **80**: 1826–1827

Office for National Statistics (1999) *Cancer statistics – registrations, England & Wales*, 1993. ONS Series MB1 No. 26. London: The Stationery Office

Parkin DM, Chen VW, Ferlay J, Galceran J, Storm H and Whelan S (1994) *Comparability and quality control in cancer registration.* IARC Technical Report No. 19. Lyons: IARC

Thames Cancer Registry (1997) *Report for 1996*. London: TCR

doi: 10.1054/ bjoc.2000.1256, available online at http://www.idealibrary.com on IDEAL®

**Sir**,

We read the letter from Quinn et al with interest and are glad to have an opportunity to acknowledge the cooperation received from the staff at the ONS throughout our study. The ONS team made considerable efforts to provide comprehensive reporting of ovarian cancer cases. Our report was not intended to be a criticism of the important work performed by the NHSCR.

We reported our comparison of ‘direct’ and NHSCR follow up for ovarian cancer in order to provide information for the design of future research studies. Although the two methods of follow up are complementary, direct follow up identified more cases of ovarian cancer and identified them in a shorter period of time than was possible via the NHSCR. Researchers need to be aware of the issues of incomplete registration and the delay in notification through the NHSCR and consider the option of using an additional method of follow up. These issues have major implications for the design of clinical trials and in this context we hope that the data provided by our study is of some value.

Quinn et al highlighted the limitations of ‘follow-up’ compared to ‘flagging’ studies via the NHSCR. Whilst these points are entirely valid they do not explain the eleven cases of ovarian cancer not identified by the NHSCR in our study. First, follow up was carried out by the NHSCR in 1997–98, a time point more than five years after diagnosis of the ovarian cancer cases in our study. Second, repeated searches were performed by the NHSCR for the eleven cases both manually and by computer. Although the data originally supplied to the NHSCR was incomplete for some study participants, complete data for the relevant eleven cases was resubmitted for additional searches once the discrepancy was identified. It is possible that flagging would eventually identify these cases but a delay of more than five years from study completion to analysis has major implications for a clinical trial. As noted by Quinn et al a total of four cases of ovarian cancer reported by the NHSCR were not identified by direct follow-up. However, the study was limited to cases diagnosed between 1986 and 1993 because this allowed a 5 year period for data collection by the NHSCR and was the period of direct follow. Two cases of ovarian cancer identified by the NHSCR but diagnosed after 1993 were not therefore reported in our paper. The same applies to three other cases of ovarian cancer diagnosed after 1993 but not identified by the NHSCR.

It seems sensible for researchers currently designing clinical studies requiring long-term follow-up to consider using direct follow up as well as flagging with the NHSCR. Direct follow up is a rapid and reliable means of identifying cancer cases which complements information provided by the NHSCR. Major efforts and numerous changes are being made in the cancer registration system which are improving the research value of this key resource. We strongly support investment in cancer registration and appreciate the efforts being made by staff in the regional cancer registries and at the ONS. Hopefully in the future the use of direct follow up in clinical trials will not be necessary!

*Nicola MacDonald, Usha Menon, Ian Jacobs*
*St Bartholomew’s and Royal London School of Medicine and Dentistry, Queen Mary and Westfield College*

*Ian Jacobs MD MRCOG Professor of Gynaecological Oncology*
*St Bartholomew’s Hospital, London EC1A 7BE*

© 2000 Cancer Research Campaign 

